# Different Histories, Different Destinies‒Impact of Evolutionary History and Population Genetic Structure on Extinction Risk of the Adriatic Spined Loaches (Genus *Cobitis*; Cypriniformes, Actinopterygii)

**DOI:** 10.1371/journal.pone.0131580

**Published:** 2015-07-15

**Authors:** Ivana Buj, Marko Ćaleta, Zoran Marčić, Radek Šanda, Jasna Vukić, Milorad Mrakovčić

**Affiliations:** 1 Department of Zoology, Faculty of Science, University of Zagreb, Zagreb, Croatia; 2 Faculty of Teacher education, University of Zagreb, Zagreb, Croatia; 3 National Museum, Prague, Czech Republic; 4 Department of Ecology, Charles University, Prague, Czech Republic; National Cheng-Kung University, TAIWAN

## Abstract

The region of Balkans is often considered as an ichthyologic “hot spot”, with a great number of species and high portion of endemics living in fresh waters in a relatively small area. The Adriatic watershed in Croatia and Herzegovina is inhabited by six spined loach species (genus *Cobitis*) whose extinction risk estimations were based solely on their extent of occurrence (and/or area of occupancy) and its fragmentation, and conservation proposals do not consider diversity below species level. In this investigation we employed molecular genetic methods to describe present genetic structure of the Adriatic spined loaches and reveal their demographic history. The divergence of the Adriatic lineages inside the genus *Cobitis* started in Miocene and lasted until Pleistocene epoch. Geological events responsible for shaping recent diversity of spined loaches in the Adriatic basin are: the Dinarid Mountains upwelling, the evolution of Dinaric Lake system, local tectonic activity, river connections during glaciations and differences in sea level. Even though all the investigated species inhabit karstic rivers located in the same geographic area and that were subject of similar geological events, the results obtained reveal great differences in their genetic diversity and structure and point out the necessity of different conservation measures to ensure their future viability. High level of genetic polymorphism is characteristic for species located more to the south. Two species comprised of more than one population have completely different intraspecific structure; populations of *C*. *illyrica* are genetically distinct and represent separate evolutionary significant units, whereas intraspecific structure of *C*. *narentana* corresponds to metapopulational pattern. Without population genetic data, evolutionary significant units could be easily misidentified. Furthermore, the obtained results affirm that population genetic measurements are able to detect differences among closely located and related species and estimate extinction risk even more accurately than currently applied IUCN criteria.

## Introduction

The International Union for Conservation of Nature and Natural resources (IUCN) recognizes the need to conserve biodiversity at three levels: genetic diversity, species and ecosystems [1]. Currently, the IUCN Red List of Threatened Species (IUCN, 2012) is the most important mechanism for classifying species based on their extinction risk [2]. Regardless of the extensive evidence pointing out that genetic factors influence extinction risk [3], [4] and growing amount of population genetic data available for endangered species, those data are included into the IUCN Red List categorization system for threatened species [5] only by extrapolation of census (*N*) from effective (*N*_*e*_) population size for criterion C (Small population size and decline). Furthermore, practical conservation efforts still focus almost exclusively on species [6], often overlooking biodiversity components below species level. Since it is not possible to protect all biodiversity features due to time and financial limitations [7], it is important to implement methods that will, in shortest time, allow most accurate estimation of the extinction risk, as well as identification of units below species level that will most likely ensure evolutionary course of the endangered species concerned.

**Fig 1:**
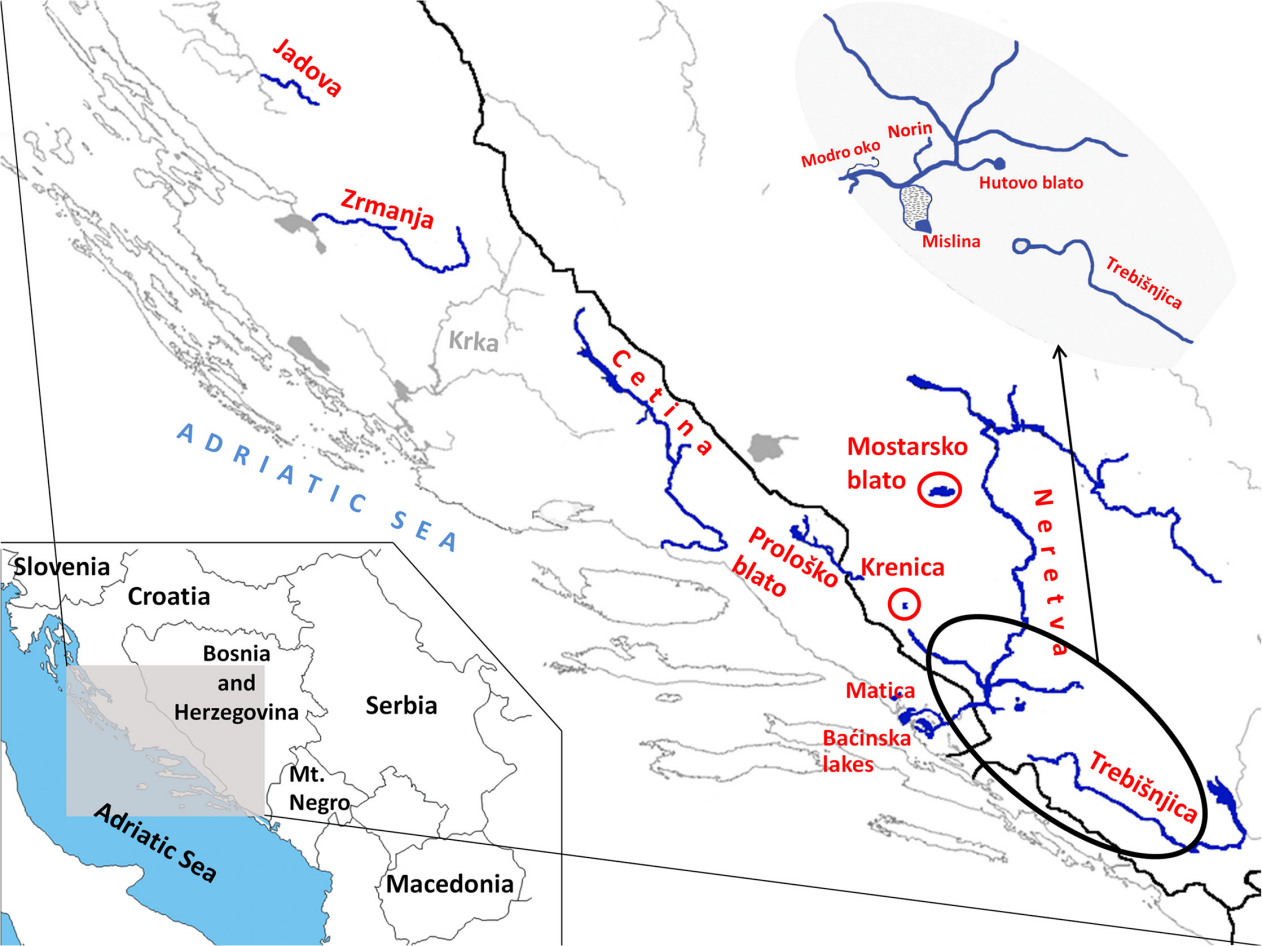
Map of investigation area. *Cobitis jadovaensis* is distributed in Jadova R., *C*. *bilineata* in Zrmanja R., *C*. *dalmatina* in Cetina R. *Cobitis narentana* lives in Neretva R., Mislina, Modro oko lake, Norin R., Trebišnjica R. and Hutovo blato wetland. *Cobitis illyrica* inhabits Matica R. with Baćinska lakes, as well as Prološko blato and Krenica lakes. *C*. *herzegoviniensis* is distributed in Mostarsko blato karstic field.

Based on investigations conducted in past years, the region of Balkans, especially the area of the Adriatic watershed in Croatia and Bosnia and Herzegovina, has been recognized as a diversity “hot spot” and a center of endemism for the European spined loaches (genus *Cobitis*) [8]. Majority of *Cobitis* species distributed in the mentioned area are considered endangered pursuant to the IUCN categories and significant anthropologic impact is posing threat to their survival. On the other hand, most of them are endemic, distributed in a single river, river basin or only on several localities in a very small area (Fig 1). *Cobitis jadovaensis* Mustafić & Mrakovčić, 2008 is endemic to small karstic river Jadova and is considered critically endangered (CR). *Cobitis bilineata* Canestrini, 1865 in Croatia inhabits the Zrmanja R., but is also distributed in Slovenia, Italy, France and Spain. It is included as the least concern species (LC) in the IUCN Red List. *Cobitis dalmatina* Karaman, 1928 inhabits a single river in the middle Dalmatia, the Cetina R., while *C*. *narentana* Karaman, 1928 is endemic to the Neretva R. basin. Those two species are estimated as vulnerable (VU). *Cobitis illyrica* Freyhof & Stelbrink, 2007 is distributed fragmentarily, inhabiting the Matica R. and Baćinska lakes, as well as lakes Prološko blato and Krenica and is considered as CR. For *C*. *herzegoviniensis* Buj & Šanda, 2014 extinction risk estimation has not yet been conducted (www.iucnredlist.org), but it lives only in the Mostarsko blato karstic field. Previous phylogenetic and taxonomic investigations revealed that *Cobitis* populations from Dalmatia and Herzegovina, together with the Italian *C*. *bilineata* samples, form so called “bilineata” subgroup inside the Adriatic phylogenetic group [8], [9]. Even though taxonomic status and phylogenetic relationships of the Adriatic spined loaches have been investigated, data on their evolutionary history are very scarce. Knowledge on population genetic structure and diversity, as well as understanding of evolutionary and demographic history of certain species has a great importance for its conservation since it enables prediction of its future trend and estimation of survival probability.

In this investigation we employed molecular genetic methods to describe present genetic structure of the endemic spined loaches in the Adriatic basin in Croatia and Bosnia and Herzegovina and reveal its evolutionary history. We aimed to answer the question whether the same geographic area and similar geological events provoked similar evolutionary course and caused alike intraspecific genetic polymorphism or were the evolutionary histories of closely located and related species different in a way that could be important for their future trend and, thereafter, conservation strategies? Current extinction risk estimation for those species was based solely on their extent of occurrence and its fragmentation. Conservation proposals do not consider diversity below species level, nor any differences among species except distribution range. On the example of the Adriatic spined loaches, we intended to test whether population genetic and evolutionary history data can enable finding finer scale differences among species, estimating their extinction risk more accurately and proposing more adequate conservation measures that would, at the same time, ensure preservation of the biodiversity below species level.

## Materials and Methods

### Ethics Statement

This investigation was conducted entirely in accordance with ethical standards and Croatian legislation. The work was approved by the Ethical Committee of the Faculty of Science, University of Zagreb.

The sample analyzed in this investigation comprises DNA sequences of individuals from all known *Cobitis* populations in the Adriatic watershed of Croatia and Bosnia and Herzegovina. Besides sequences used in taxonomic investigation of the Adriatic spined loaches [8], the sample was completed with two new locations and more sequences from populations that were represented with fewer samples in the mentioned investigation (Table 1). Above mentioned taxonomic investigation was based on various methods of phylogenetic reconstruction, morphological analyses, as well as comparison of phylogenetic models using Bayes factors [8] whereas in this investigation we have analyzed evolutionary history and population genetic structure using population genetic methods. Three genetic markers were analyzed: mitochondrial gene for cytochrome *b* (cyt *b*) and two nuclear genes: RAG1 gene and the first intron of the S7 gene, even though all three markers were not included in all analyses. Namely, different position and function of investigated markers, as well as their evolution inside the genus *Cobitis*, result in different credibility of phylogenetic and population genetic analyses. Cyt *b* gene, as a part of mitochondrial DNA (mtDNA), is inherited only maternally, has a faster mutational rate, lacks recombination and other mechanisms that would effectively erase mutations, and, thereafter, is phylogenetically more informative. RAG1 and S7 are nuclear genes, but inside S7 non-coding intron was analyzed, whereas RAG1 is a protein-coding gene. Since it was concluded that cyt *b* phylogenetic tree mostly resembles species diversification tree [8] and the only known mutation rate inside spined loaches, available for the molecular clock calibration, is the one of the cyt *b* gene, phylogenetic reconstruction, as well as divergence times and past changes in population size estimations were conducted based on cyt *b* data set. On the other hand, tests of genetic diversity and differentiation, as well as estimation of the gene flow among populations were based on all three genes in order to obtain the most reliable results based on both, mitochondrial and nuclear DNA, and reveal eventual differences. Effective population size estimations were conducted on cyt *b* data set, because the knowledge on mutation rate is also necessary for those calculations.

**Table 1:** Sampling localities and number of specimens included in the investigation.

species	locality	coordinates	No of samples
*C*. *jadovaensis*	Jadova R.	44.5031, 15.5458	8
*C*. *bilineata*	Zrmanja R.	44.1923, 15.7857	18
*C*. *dalmatina*	Cetina R. (Blato)	43.4806, 16.8423	18
	Vinalić	43.9374, 16.4297	2
*C*. *narentana*	Neretva R. in Metković	43.4806, 16.8423	3
	Mislina channel	42.9923, 17.6153	9
	Norin	43.0803, 17.6288	15
	Modro oko lake	43.0575, 17.5102	14
	Trebišnjica R.	42.8682, 17.9778	6
	Hutovo blato	43.0418, 17.7452	4
*C*. *illyrica*	Prološko blato	43.4749, 17.122	8
	Matica	43.1752, 17.3865	10
	Krenica	43.3742, 17.331	10
	Rastočko field	43.2081, 17.3992	2
*C*. *herzegoviniensis*	Lištica R.	43.3247, 17.7367	11

Laboratory protocols for the DNA extraction, polymerase-chain reaction (PCR) and sequencing for new samples were the same as for samples used in taxonomic investigation and are described in [8]. Haplotype variants of nuclear genes in heterozygous individuals were reconstructed by a Bayesian statistical method implemented in PHASE 2.1 software [10], [11]. Phylogenetic reconstruction was conducted using Bayesian inference as implemented in MrBayes (version 3.1.2 [12]). Besides our sequences of cyt *b* gene, in the phylogenetic reconstruction and divergence time estimation we have also included haplotypes of *C*. *elongata* Heckel & Kner, 1858 and Italian *C*. *bilineata* [9], retrieved from the GenBank. The sequences of *C*. *elongatoides* Baçescu & Maier, 1969 and *Sabanejewia romanica* (Baçescu, 1943) [13] were used as outgroups. Two simultaneous runs were conducted. For each, Markov Chain Monte Carlo was run four times for three million generations with trees sampled every 100 generations. The best-fitting model of molecular evolution (GTR model) was selected by hierarchical likelihood ratio tests using MODELTEST software version 3.06 [14]. Average standard deviation of split frequencies approached 0 (0.000074) and the potential scale reduction factor approached 1 (1.002). Convergence between runs was high for all trace parameters, as investigated using Tracer v1.5.0 (software by Rambaut A, Suchard MA, Xie D & Drummond AJ; available from http://beast.bio.ed.ac.uk/Tracer). Divergence times between investigated species, as well as between phylogenetic lineages, were estimated by a Bayesian MCMC coalescent method, using Beast 1.7.0 software [15]. The rate homogeneity across phylogenetic lineages was assessed by the log-likelihood ratio test (LRT), comparing the likelihood of phylogenetic trees (reconstructed using maximum likelihood approach) with and without molecular clock enforcement in PAUP* (version 4.0b10 [16]). Since the likelihood scores were the same in both cases, we applied a strict molecular clock. Branch rates were drawn from an uncorrelated lognormal distribution and a Yule speciation prior with random starting tree. Substitution model applied was HKY with Gamma site heterogeneity model. We used default prior distributions for kappa, frequencies and alpha, whereas substitution rate parameters were unlinked across codon positions. The number of MCMC steps (the length of chain) was three millions. The molecular clock calibration was conducted based on the divergence rate of cyt *b* gene of 0.34% per lineage per million years (0.68% per pairwise divergence [17]). Phylogenetic reconstruction and divergence time estimation was based on cyt *b* data set only due to differences in three gene trees and conclusions of [8] that cyt *b* phylogenetic tree mostly resembles species diversification tree. Upon divergence time estimations based on the cyt *b* gene, we have used two estimated splitting-events as calibration points to assess the divergence rates of RAG1 and S7 first intron.

The level of intraspecific and intrapopulational genetic diversity was estimated by calculating several measures of DNA polymorphism for each genetic marker, using DnaSP v.5 [18]: number of polymorphic sites (S), haplotype diversity (Hd), average number of nucleotide differences (K) and nucleotide diversity (π). Furthermore, a frequency of each haplotype was calculated as a percentage of a certain haplotype in a population. For species distributed on more than one locality and presumably comprising more than one population (*C*. *narentana* and *C*. *illyrica*) intrapopulational genetic diversity was calculated for each population. Genetic differentiation analysis was employed in order to explore differences among populations of the same species. It comprised estimation of haplotype‒based statistics (χ^2^ test, H_ST_), as well as nucleotide sequence‒based statistics (K_ST_, K_ST_*, K_S_*, Z, Z*) [19] and was conducted on all three investigated genes. The statistical significance of each statistic was ascertained by a permutation test. Changes in past effective population sizes were analyzed using Bayesian skyline plots (BSP), as implemented in Beast 1.7.0. The BSP model was employed on cyt *b* data set and it estimates the history of change in effective population size from the variability among sampled haplotypes, assuming a mutation rate of 0.34%/MY. The BSP settings were the same as used for divergence time estimations with the tree prior set to Coalescent: Bayesian Skyline.

Interactions among populations and species were investigated using maximum likelihood approach [20], [21] implemented in MIGRATE 3.2.1 [22]. We have calculated immigration rates between populations of species containing more than one population as mutation-scaled effective immigration rates and as the number of immigrants per generation. Migration rates were calculated separately for mitochondrial cyt *b* gene and nuclear data set (combined RAG1 and S7 first intron), due to the differences in the evolution of mitochondrial vs. nuclear DNA. Regarding nuclear data set, MIGRATE calculates two loci estimates independently, and multi-locus estimate takes into account the likelihood of each parameter at each locus. Same software was employed for the effective population sizes (*Ne*) estimations. Extrapolation of census population size (*N*) was based on formula *N*_*e*_/*N* = 0.1–0.2 [23]. Since exact *N*_*e*_/*N* value has not been estimated for spined loaches, we used current default value only to enable comparison with IUCN criterion C thresholds, taking into account drawbacks of such extrapolation [23]. Data on genetic diversity (intrapopulational) and differentiation (among populations) as well as interactions among populations of the same species, even though are not usually considered in extinction risk assessments, might be of great assistance in species’ future trend predictions and conservation decisions.

## Results

GenBank accession numbers of cyt *b* sequences are: EF605302-EF605306, EF605312-EF605316, KJ487435-KJ487484, KP208162-208173; of RAG1 haplotypes: KJ487485-KJ487519; and of S7 first intron: KJ487520-KJ487554.

**Fig 2:**
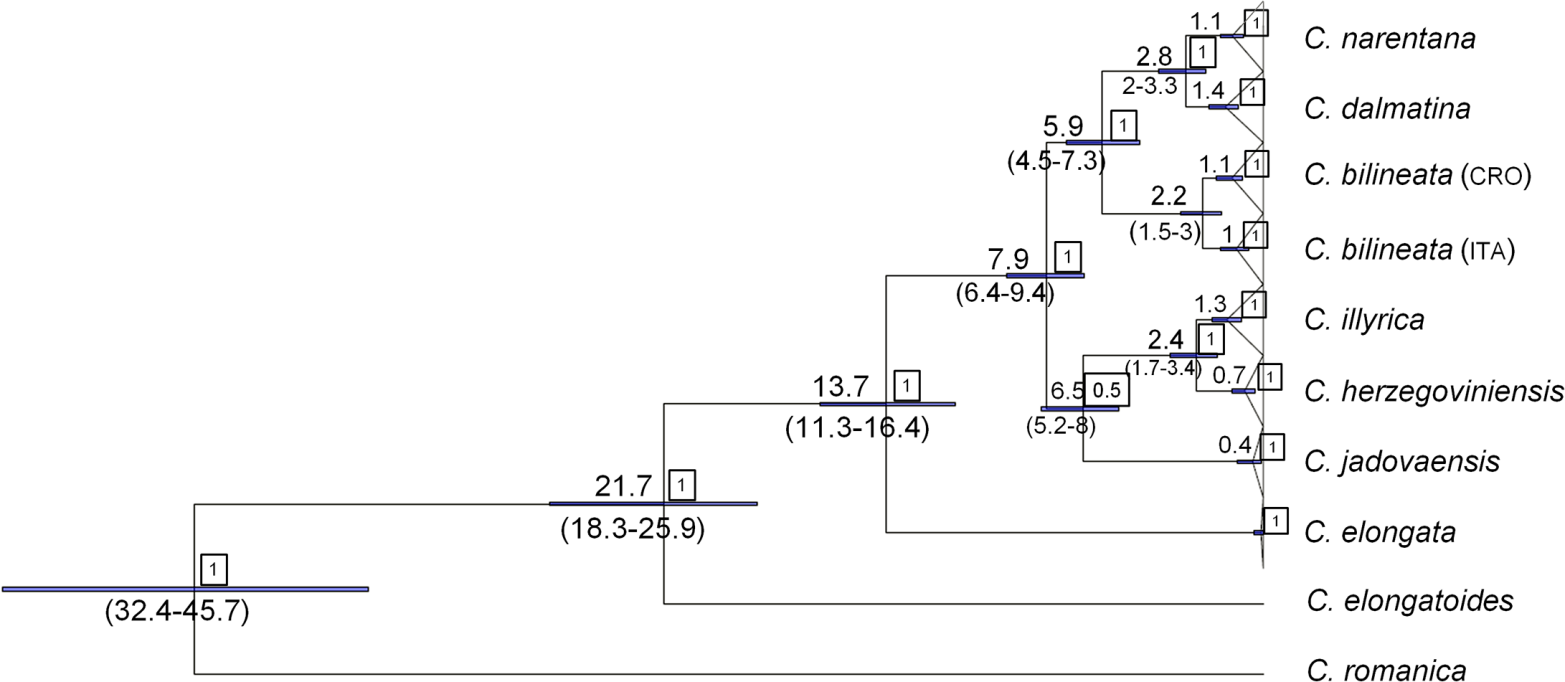
Divergence time estimations based on the cyt *b* sequences of the Adriatic spined loaches. Numbers in squares represent BI posterior probabilities. Timing of the splitting events is presented by mean value and the 95% credibility range (in million years ago).

Phylogenetic tree obtained by Bayesian inference with timing of the splitting events is presented on the Fig 2. Phylogenetic position of all species and lineages was the same as revealed by previous taxonomic investigation [8] in which also other methods of phylogenetic reconstruction, as well as comparison of phylogenetic hypotheses was conducted. The divergence of the Adriatic lineages inside the genus *Cobitis* started in Miocene and lasted until Pleistocene epoch. Mutation rates of two investigated nuclear genes were similar and about six times slower than of the cyt *b* gene (0.10 and 0.12% per pairwise divergence for RAG1 and S7 first intron, respectively).

Table 2 summarizes measures of genetic polymorphism for all species. Taking into account all three investigated genetic markers (but considering cyt *b* to be the most reliable in elucidating intraspecific and intrapopulational genetic diversity due to its faster mutation rate) high level of genetic polymorphism is characteristic for three species distributed more to the south (*C*. *dalmatina*, *C*. *narentana* and *C*. *illyrica*). On the other hand, especially *C*. *jadovaensis*, but then also *C*. *bilineata* express much lower level of genetic polymorphism. *Cobitis herzegoviniensis*, even though geographically belonging to the “southern group”, is characterized by lower level of genetic diversity. The frequency of haplotypes is also quite different among species. In *C*. *herzegoviniensis*, and even more pronounced in *C*. *jadovaensis* and *C*. *bilineata*, one cyt *b* haplotype predominates greatly, whereas the remaining haplotypes are found only individually. Frequencies of cyt *b* haplotypes were more equally distributed in the remaining species. Frequency of nuclear haplotypes mostly follows the same pattern, but differences among species are less pronounced and some exceptions are present. The problem with nuclear haplotypes is also that closely related species *C*. *illyrica* and *C*. *herzegoviniensis* even share some same nuclear haplotypes, most likely due to incomplete lineage sorting [8].

**Table 2:** Genetic polymorphism of the investigated species based on three genetic markers.

species	N	h	S	Hd	K	π	f (%)
	cyt *b*						
*C*. *jadovaensis*	8	2	2	0.250	0.500	0.00044	JAD1:87.5, JAD2:12.5
*C*. *bilineata*	18	5	9	0.405	1.098	0.00096	ZRM1: 77.8, ZRM2-5:5.6
*C*. *dalmatina*	20	18	26	0.990	3.816	0.00335	5–10
*C*. *narentana*	51	24	28	0.948	3.296	0.00289	2–11.8
*C*. *illyrica*	31	18	23	0.946	4.280	0.00375	3.2–16.1
*C*. *herzegoviniensis*	11	6	6	0.727	1.236	0.00108	HER1: 54.6, HER2-6: 9,1
	RAG1						
*C*. *jadovaensis*	12	2	1	0.409	0.409	0.00045	rJAD1:83.3, rJAD2:16.7
*C*. *bilineata*	26	3	2	0.218	0.225	0.00025	rZRM1:88.5, rZRM2:7.7,rZRM3:3.9
*C*. *dalmatina*	26	7	5	0.726	1.065	0.00117	3.9–46.2
*C*. *narentana*	82	8	7	0.311	0.403	0.00044	rNER1:83.3, rNER2-8:1.1–6.7
*C*. *illyrica*	44	14	9	0.887	2.896	0.00318	1.9–32.7
*C*. *herzegoviniensis*	8	3	4	0.607	1.536	0.00169	12.5–62.5
	S7 first intron						
*C*. *jadovaensis*	10	4	5	0.778	2.356	0.00463	10–40
*C*. *bilineata*	10	2	2	0.467	0.933	0.00183	sZRM1:70, s ZRM2:30
*C*. *dalmatina*	8	7	13	0.964	4.893	0.00961	12.5–25
*C*. *narentana*	34	14	16	0.799	1.627	0.00319	2.5–37.5
*C*. *illyrica*	30	9	10	0.632	1.313	0.00258	sILL1:56.3, sILL2-10:3.1–15.6
*C*. *herzegoviniensis*	8	2	1	0.25	0.25	0.00049	sILL1:87.5, sHER1:12.5

N–number of sequences; h–number of haplotypes; S–number of polymorphic sites; Hd–haplotype diversity; K–average number of nucleotide differences; π –nucleotide diversity; f–haplotype frequency.

**Fig 3:**
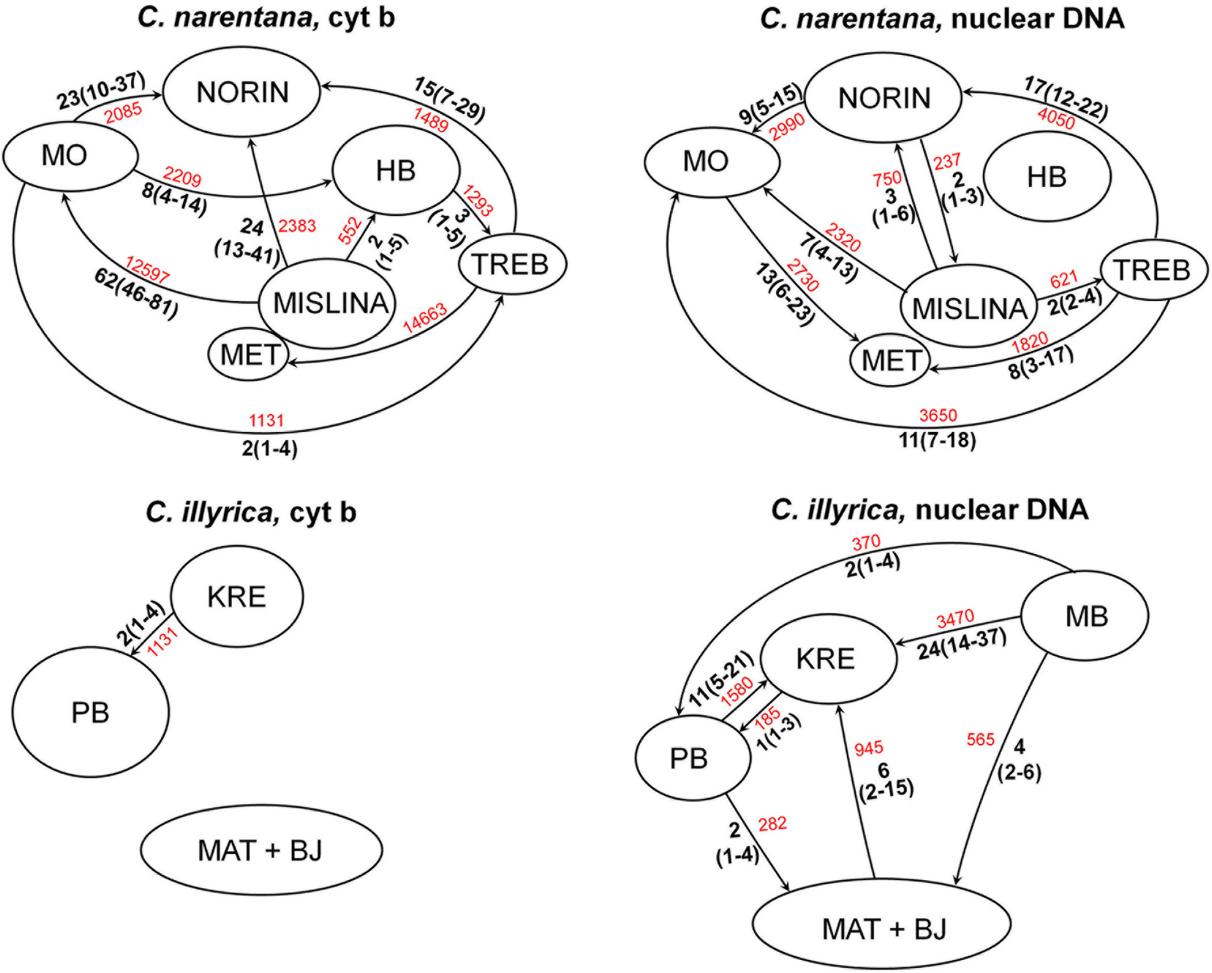
Migration rates among populations of *C*. *narentana* and *C*. *illyrica* based on mitochondrial (cyt *b*) and nuclear genes (RAG 1 and S7 first intron). Bold numbers present maximum likelihood estimation of the number of immigrants per generation (90% credibility range is presented in the parenthesis), whereas regular numbers are mutation-scaled effective immigration rates. Arrows indicate migration direction.

For species found on more than two localities, in order to conceive adequate conservation measures it was important to find out if the populations on different localities genetically differ and what amount of migration and gene flow is present among them. The results of genetic differentiation tests are completely different for *C*. *narentana* and *C*. *illyrica*, despite immediate proximity of their distribution ranges (Fig 1). There is no genetic differentiation among *C*. *narentana* populations (out of all calculated statistics for three markers only K_ST_ for cyt *b* data set turned out to be statistically significant), whereas three populations of *C*. *illyrica* are indeed genetically different (all values statistically significant with the exception of χ^2^ for the S7 first intron). That conclusion is also corroborated by the Migrate results (Fig 3). All *C*. *narentana* populations are interconnected with migrations based on both cyt *b*, as well as nuclear markers. On the other hand, cyt *b* indicates presence of very restricted migrations only among Krenica and Prološko blato populations of *C*. *illyrica*. The third *C*. *illyrica* population (distributed in the Matica R. and Baćinska lakes) does not seem to be connected with other populations based on mtDNA. Nuclear genes, however, evinced more pronounced migrations among all *C*. *illyrica* populations, as well as *C*. *herzegoviniensis* from Mostarsko blato.

The level of intrapopulational genetic diversity for populations of *C*. *narentana* and *C*. *illyrica* (Table 3) differs among populations and also points out differences between this two species that should be important for conservation decisions. Inside *C*. *narentana* highest levels of genetic polymorphism is characteristic for populations from Modro oko and Norin, whereas among *C*. *illyrica* populations Krenica has the greatest amount of genetic diversity. Nevertheless, all populations of this two species express high genetic polymorphism.

**Table 3:** Genetic polymorphism revealed inside populations of *C*. *narentana* and *C*. *illyrica* based on cyt *b*/RAG 1/S7 first intron.

	N	h	S	Hd	K	Pi
*C*. *narentana*						
Metković	3/6/6	3/1/4	4/0/6	1/0/0.8	2.667/0/2	0.002/0/0.004
Mislina	9/18/8	6/5/5	9/4/5	0.889/0.484/0.786	3.778/0.641/1.250	0.003/0/0.003
Modro oko	12/26/8	8/5/5	14/5/6	0.894/0.351/0.786	3.242/0.455/1.5	0.003/0/0.003
Norin	15/24/4	14/4/4	20/4/6	0.991/0.308/1.000	3.771/0.409/3.167	0.003/0/0.006
Trebišnjica	6/8/8	4/1/4	4/0/4	0.800/0/0.750	1.867/0/1.179	0.002/0/0.002
Hutovo blato	4/-/-	3/-/-	3/-/-	0.833/-/-	1.5/-/-	0.001/-/-
*C*. *illyrica*						
Matica	10/20/10	6/4/5	12/4/6	0.778/0.658/0.844	2.867/1.526/2	0.003/0.002/0.004
Prološko blato	8/14/10	4/7/3	3/6/4	0.750/0.758/0.378	1.036/2.253/1.111	0.001/0.003/0.002
Krenica	10/10/10	7/7/4	8/7/3	0.933/0.911/0.533	2.400/2.556/0.6	0.002/0.003/0.001

N–number of sequences; h–number of haplotypes; S–number of polymorphic sites; Hd–haplotype diversity; K–average number of nucleotide differences; π –nucleotide diversity.

**Fig 4:**
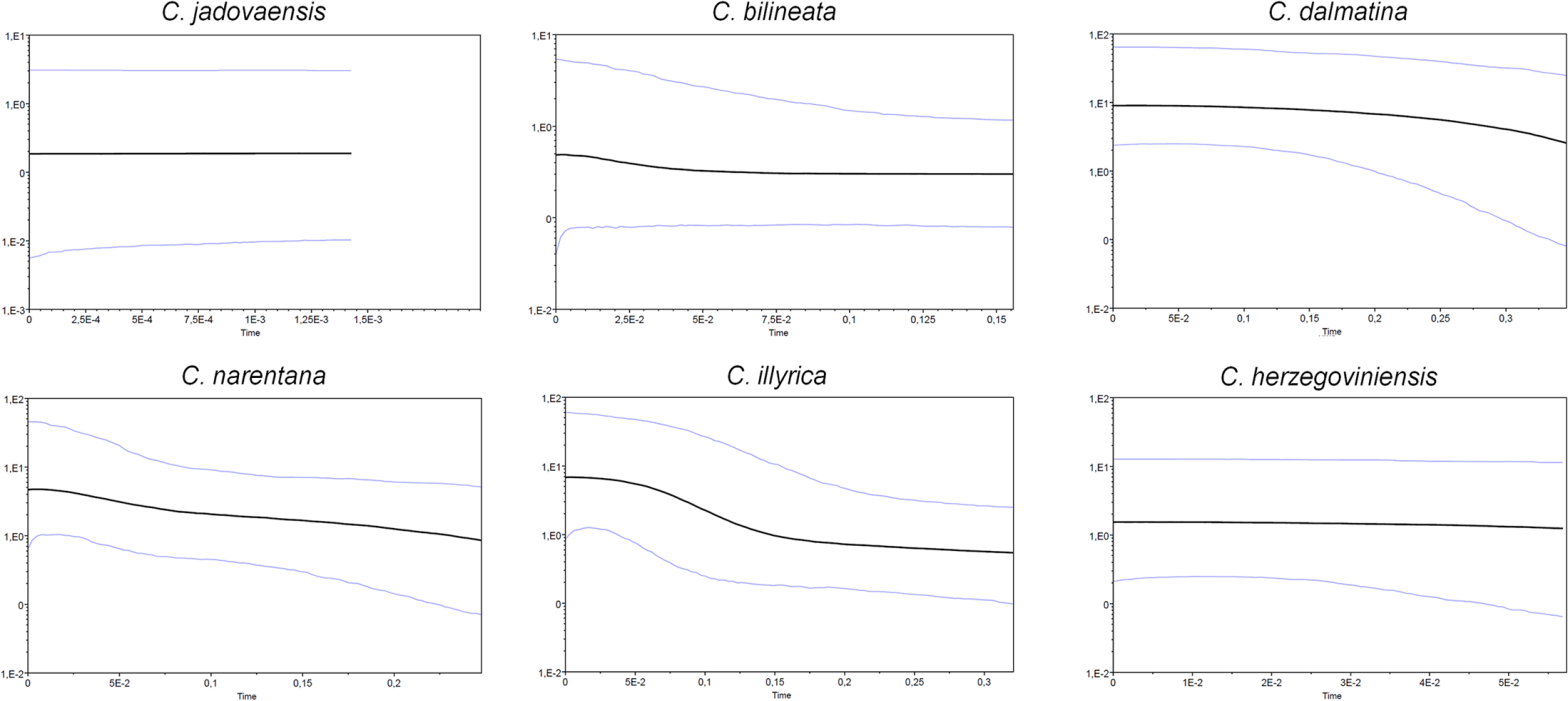
Bayesian skyline plots based on cyt *b* sequences. Changes in effective population size (millions of individuals on a log scale; Y-axes) are depicted over time (in million years; X-axes). Black central lines represent the median values of Ne, while blue lines represent the 95% highest posterior density of the Ne estimates.

Bayesian skyline plots (Fig 4) demonstrated population growth for *C*. *dalmatina* and *C*. *narentana*, but they were able to explain only more recent period of demographic history (from 0.3 MYA), whereas based on Bayesian MCMC coalescent method (with molecular clock enforcement) it seems that intraspecific divergence of those species started much earlier. In the final stage (20000 YA till present) of the demographic history of *C*. *bilineata* slight growth can also be noticed on BSP, preceded by a stabile population stage from 150000 YA. For *C*. *jadovaensis* only last 15000 years were explained on BSP, and for that period population seems to be stable. *Cobitis herzegoviniensis* is also estimated to have stable effective population size in the most recent period of its demographic history.

In accordance to the genetic polymorphism measures, effective population sizes also differ among species and populations (Table 4) and vary between 458 estimated for *C*. *jadovaensis* to 12316 in *C*. *narentana*. Even though effective population size estimations with 50% probability, and especially with 90% probability, resulted in greater ranges of values, they enabled identification of conservation priorities. For the species with the smallest effective population size (*C*. *jadovaensis*) even the highest estimation (at 95^th^ percentile) is under the threshold of 1000 [19].

**Table 4:** Effective population sizes (*N*_*e*_) and extrapolated census population sizes (*N*) based on *N*_*e*_/*N* = 0.1–0.2.

Species (population)	*N*_*e*_ (MLE)	*N*_*e*_ (25–75% prob.)	*N*_*e*_ (5–95% prob.)	*N*
*C*. *jadovaensis*	458	359–600	244–867	2290–4580
*C*. *bilineata* (Zrmanja)	2288	1891–2629	1526–3406	11440–22880
*C*. *dalmatina*	6252	5352–7364	4341–9465	31260–62520
*C*. *narentana* (Norin)	4000	2783–5960	1388-*	20000–40000
*C*. *narentana* (Modro oko)	2900	1810–4731	1063-*	14500–29000
*C*. *narentana* (Mislina)	3241	2851–3491	2480–4080	16205–32410
*C*. *narentana* (Hutovo blato)	2176	1398–3653	*	10880–21760
*C*. *narentana* (Trebišnjica)	1200	874–1600	606–2691	6000–12000
*C*. *illyrica* (Matica and Baćinska lakes)	1582	1205–2011	874–3080	7910–15820
*C*. *illyrica* (Prološko blato)	1064	811–1348	589–2069	5320–10640
*C*. *illyrica* (Krenica)	2358	1966–2697	1594–3469	11790–23580
*C*. *herzegoviniensis*	1123	891–1366	680–1931	5615–11230

For effective population size values, maximum likelihood estimation (MLE), as well as profile likelihoods at different percentiles (25–75% and 5–95% probabilities) are presented. Census population sizes were extrapolated using maximum likelihood estimation of effective population size for each population.

*The convergence to the percentile value was not successful.

## Discussion

Even though all investigated spined loach species inhabit karstic rivers located in the same geographic area and that were subject to similar historical geological events, results of this investigation reveal great differences in their genetic diversity and structure. Based on this data it is clear that same conservation measures cannot be adequate for all investigated species, just as their real status differs.

### Evolutionary History of the Adriatic Spined Loaches

Time frames of important divergence events among the investigated Adriatic spined loaches correspond to recorded geological events. Separation of the “bilineata” subgroup from the ancestor that it shared with *C*. *elongata* (distributed in the Danube R. basin) occurred in the middle Miocene (11.3–16.4 MYA), which is concordant with the Dinarid Mountains uplift. The next evolutionary event characterized separation of main lineages inside the “bilineata” subgroup, which is dated to the second half of Miocene and possibly beginning of Pliocene. It is likely that separation and evolution of main groups and lineages of the Adriatic spined loaches was shaped by the evolution of Dinaric Lake system (DLS) and local tectonic activity. DLS is a system of tectonic lakes filled with freshwater and located inside the western Dinaric belt [24], [25]. It originated in the early Miocene and during its evolution lakes changed sizes and were temporary interconnected [26], [27]. Fossil evidence indicates that lakes belonging to DLS were inhabited by freshwater, highly endemic fauna [25], [28]. Even though fossil records of fish species from DLS were never investigated, our results reveal the main older diversification events inside spined loaches to also be connected with the so called spectacular Miocene radiation [28]. In accordance with consideration that DLS was a root for the evolution of snail genera *Orygoceras*, *Emmericia* and *Melanoptychia* [28], it is likely that evolution of DLS during favorable Miocene climatic conditions enabled diversification of cyprinid fishes. Nevertheless, diversification of the Adriatic spined loaches did not come to an end with the termination of DLS in lower Miocene or early Pliocene. The next important evolutionary event, represented by the origination of the majority of recent Adriatic species can be dated back to the period of the lower Pliocene and the beginning of the Pleistocene (1.5–3.4 MYA). There is geological evidence that tectonic activity in the investigated area was especially intense on the Pliocene/Pleistocene boundary, so it is likely that local tectonic events induced origination of the majority of the recent *Cobitis* species. It is interesting that even after that period a contact between the Zrmanja and the Italian *C*. *bilineata* populations was possible, resulting in the presence of the same haplotypes inside two isolated populations. Namely, the Po R. and the Zrmanja R. were periodically connected until the raising of the sea level in Holocene. Connections were formed during glaciations when sea level was much lower and the area of northern Adriatic desiccated [29]. Geological evidence demonstrated that historical Neretva and Cetina R. beds were significantly longer than recent [30], [31]. Based on our results, those two rivers not only had significantly longer beds, but were interconnected (constantly or periodically) until lower Pliocene (about 2.8 MYA). That is the period of the Pliocene optimum, with very warm climate conditions and sea level higher for 20–35 m comparing to recent conditions [32]. High sea level could have led to the final isolation of two rivers and provoked differentiation of two *Cobitis* species, even though it is also possible that intensive tectonic activity played a role in this segregation. Separation of *C*. *illyrica* and *C*. *herzegoviniensis* occurred concurrently and it could also be provoked by local tectonic activity.

Glaciations, that started in the final stage of Pliocene (Gelasina, 2.58 MYA [32]) further affected evolutionary history of the Adriatic spined loaches, most likely not as a diversification event, but by influencing on demographic history of species (as will be discussed later).

### Intraspecific Genetic Diversity–Causes and Consequences

Pronounced differences among geographically closely located and phylogenetically related species in present pattern of DNA polymorphism imply different demographic history of each species after their separation from the common ancestor, but also points out different probabilities of viability and future survival.

Species *C*. *jadovaensis* contains extremely low genetic diversity, which together with skewed allele frequency distribution (great domination of one haplotype) is an indication of a strong evolutionary bottleneck. Therefore, even though *C*. *jadovaensis* is an old species (probably of the middle Pliocene origin), its intraspecific genetic diversity, that serves as a reservoir for coping with potential future environmental changes, was significantly reduced during an evolutionary bottleneck, which also reduces its viability potential and points out necessity of effective protection. Maximum likelihood estimation of its effective population size is even smaller than previously proposed *N*_e_ of 500 (50/500 rule [33]) and more recently revised *N*_e_ ≥ 1000 [26] that is considered sufficient to maintain the evolutionary potential.

Besides in *C*. *jadovaensis*, the lowest genetic diversity was noticed in Croatian population of *C*. *bilineata* and in *C*. *herzegoviniensis*. Observed haplotype frequency also implies the possibility of a bottleneck in an evolutionary history of spined loaches from the Zrmanja R. The bottleneck hypothesis could not be confirmed using estimations of BSPs because the possible bottleneck happened earlier in demographic history than caught by BSPs. Nevertheless, geological investigations provided evidences that region of northern Adriatic basin was affected by glaciations [34]. Furthermore, the same investigations did not reveal traces of glaciations in southern Dalmatia, which probably allowed unconstrained development of southern populations resulting in their recent, much higher genetic diversity. It is very interesting that diversity of phenotypes of *C*. *jadovaensis* and *C*. *bilineata* is also much smaller than for the species located slightly more to the south [8].

For *C*. *jadovaensis* and Croatian population of *C*. *bilineata* BSPs imply population stability and slight growth, respectively, in the most recent period of their demographic histories which is encouraging in conservational sense. Nevertheless, earlier evolutionary events reduced their genetic diversity and effective population sizes, making them extremely vulnerable in case of changed environmental conditions.

All measures of intraspecific cyt *b* polymorphism are also very small for *C*. *herzegoviniensis*, even though it is located further to the south. However, its lower intraspecific polymorphism is most likely a result of its younger divergence, as well as very narrow distribution on a small, unique area.

The pattern of DNA polymorphism observed in *C*. *dalmatina* (high level of DNA polymorphism, great number of haplotypes with similar frequencies), as well as timing of the onset of its intraspecific diversity imply that *C*. *dalmatina* was represented with a large population through longer time, even in periods that for geographically closely located *C*. *bilineata* and *C*. *jadovaensis* represented unfavorable conditions and it is likely that the strong selective pressure did not affect this species. At contrary, based on MSP, we can conclude that *C*. *dalmatina* underwent a population growth.

Besides unconstrained evolutionary development, distribution on greater number of localities is probably a cause of high genetic diversity of *C*. *narentana*. In understanding the evolutionary course of *C*. *narentana* and defining conservation priorities, it was important to find out whether samples from different localities represent populations that are genetically distinct. The genetic differentiation analysis, however, revealed that Neretvanian populations are not genetically distinct. Due to smaller sample size, the power of genetic differentiation analysis is questionable, so results should be observed with caution. Nevertheless, they are corroborated by the fact that several haplotypes (of all three investigated genes) were found on 2–5 locations and that all *C*. *narentana* populations are interconnected with migration events. It is interesting, however, that immigration rates and migration directions are not the same based on mitochondrial and nuclear DNA. Such locus-specific differences in gene flow could be explained by divergent selection [35] or smaller average coalescence time and maternal inheritance of mtDNA [36], but they could also be due to possible low power of performed analyses Even if the migration rates estimated in this investigation are not completely precise, *Cobitis narentana* is an excellent example of the importance of detecting such small modifications in intraspecific structure for adequate protection of a species. Namely, without knowledge on rate and direction of migrations, it would be reasonable to propose populations from Norin and Modro oko as conservation units with an argument that they contain the highest portion of the genetic diversity of the whole species and, thereafter, will most likely ensure its survival during modified conditions. However, detailed inspection of species structure implies that the Norin population is in fact a sink population receiving immigrants from three different populations (which probably caused its higher intrapopulational diversity). Protection of sink population is only effective through protection of source populations. Better candidates for higher protection regime are populations from Modro oko and Mislina. Namely, those populations also contain high level of genetic polymorphism and great number of haplotypes, but are sources of immigrants. Even though our data corroborate stability of *C*. *narentana*, reduction of size and diversity of Modro oko and Mislina populations, and especially their disappearance, would probably have negative effect on all other populations and significantly reduce probability of survival of the whole species, even if other populations remain undisturbed. Nevertheless, the most efficient conservation strategy for ensuring *C*. *narentana* survival would include conservation of the whole metapopulation, especially since we have estimated gene flow rates based on one mitochondrial and two nuclear genes. Including more genes into further investigations would enable more accurate estimations of the gene flow and intraspecific structure, but also finding finer scale intragenomic differences.

A completely different situation was recorded for *C*. *illyrica*. Contrary to Cetina and Neretva species, the high level of intraspecific genetic diversity of *C*. *illyrica* is primarily consequence of its splitting into three geographically distinct populations: Prološko blato, Krenica and Matica with Baćinska lakes. Based on the genetic differentiation analysis, those three populations are indeed genetically different. Furthermore, with the exception of one haplotype that was found in two populations, each population has its own cyt *b* haplotypes. Two hypotheses could explain structure of *C*. *illyrica*: simultaneous colonization of localities from a single population and their subsequent isolation, or fragmentation of historical population. In any case, historical connections that allowed migrations among populations are likely to have existed. Estimation of migration rates based on nuclear genes noticed connections among all three *C*. *illyrica* populations, as well as immigrations from Mostarsko blato. On the other hand, estimation based on mitochondrial DNA found restricted immigrations only from Krenica to Prološko blato population and absence of connections among any other populations. Contrary to situation with *C*. *narentana*, in order to ensure undisturbed evolutionary development of *C*. *illyrica* it is necessary to preserve all three populations as different entities with possibly independent future evolution. *Cobitis illyrica* is just another example that present taxonomy does not completely reflect biological diversity and that species are units restricted in space, but also in time.

Based on the definition of Evolutionary Significant Unit (ESU) of Fraser and Bernatchez [37], that ESU is a lineage demonstrating highly restricted gene flow from other such lineages within the higher organizational level of the species, three populations of *C*. *illyrica* should be defined and protected as ESUs, but among *C*. *narentana* populations we could not select ESUs. The same is with Management Units (MUs; defined by Waples [38] and Moritz [39] as populations connected by little or no contemporary gene flow, but not separated historically for very long periods of time), as well as other defined conservation units. Nevertheless, due to limited resources for species comprised out of more than one population it is important to decide whether some populations are more important for future survival of the whole species. Thereafter, for conservation purposes we find more adequate the original definition of Ryder [40], that ESU is a subset of more inclusive entity species, which possess genetic attributes significant for the present and future generation of the species in question. Units below species level that are of exceptional importance for future evolution and survival of the species in question, that may or may not be genetically different from other units (lineages, populations, subpopulations) should be recognized as conservation priorities. On the example of *C*. *narentana* it is clear that two populations actually represent “the important building blocks within the species” whose maintenance is necessary to keep the process of evolution not excessively constrained [41], even though they are not genetically distinct nor substantially reproductively isolated from other conspecific populations.

### Conservation Priorities

Threats that can lead to an extinction of a certain species can be divided into two categories: anthropologic impacts (habitat loss and fragmentation, pollution, over-exploitation, introductions and translocations) and stochastic events associated with small population size [42]. Two types of extinction threats often interact causing an extinction vortex [43]. Considering future survival of the investigated spined loaches, it is fortunate that two types of extinction threats are not (yet) interacting in none of the species, e.g. species with small population sizes are under lesser human impact (*C*. *jadovaensis*, *C*. *herzegoviniensis*), whereas anthropologic impact is intense on species with greater population sizes (*C*. *bilineata*, *C*. *dalmatina* and *C*. *narentana*). Frankham et al. [23] concluded that long-term viability of populations with *N*_*e*_ <1000 is reduced because of the erosion of their ability to cope with environmental changes. Besides to *C*. *jadovaensis*, we believe that special conservation attention should be devoted to *C*. *herzegoviniensis* (given its extremely small distribution range, small genetic diversity, estimated effective population size close to 1000, and planned anthropological activities on its locality), but also to Prološko blato population. Even though *C*. *illyrica* does not seem to be endangered based on genetic criteria (estimated census size of the whole species between 25020 and 50040, BSP implying demographic growth), our results reveal separate evolutionary course of three populations comprised under that species. Effective population size of Prološko blato population is also close to 1000 pointing out greater risk of its long term survival. Even though maximum estimations of the effective population sizes of *C*. *herzegoviniensis* and *C*. *illyrica* in Prološko blato (profile likelihood at 95^th^ percentile) are larger (1931 and 2069, respectively) we still find it justified to request for special conservation attention because even those highest estimations, combined with small genetic diversity (especially in *C*. *herzegoviniensis*) do not guaranty population survival in case of planned anthropologic impact. Zrmanja population of *C*. *bilineata* also fulfils criteria to be recognized as an ESU. Due to its very small intrapopulational genetic polymorphism it should receive immediate and effective protection, even though whole species is considered LC and extrapolated census population size of Zrmanja population is higher than 10000.

### Conclusions and Recommendations

The IUCN Red List of Threatened species, produced by the Species Survival Commission (SSC) of the World Conservation Union (IUCN; http://www.iucn.org), is considered as the most comprehensive resource detailing the global conservation status of plants and animals [2] and main initiator for many national and regional conservation actions [44], [45]. On the other hand, several authors have questioned its true value in understanding patterns of, and threats to, biodiversity [2], [46–48]. Our results confirm that molecular studies describing intraspecific genetic structure, as well as patterns of gene flow and evolutionary history of a species are fundamental to effective conservation efforts [49].

Considering that population mean genetic diversity is usually positively correlated with population size, but also with mean fitness [50], it is obvious that species with lower genetic diversity have less chance of future survival not only due to smaller effective population size, but also because of smaller capacity to adopt to changed environmental conditions. In that respect, *C*. *jadovaensis* seems to be in the worst position among all Adriatic spined loaches.

Our results corroborate usefulness of the effective population size, intraspecific and intrapopulational polymorphism measures and migration rates estimations for extinction risk assessments and conservation prioritizations. Population genetic measurements are able to detect differences among closely located and related species and estimate extinction risk even more accurately than current IUCN criteria. Furthermore, those measures can be obtained much faster than demographic parameters and with minimal population disturbance (only small tissue samples are needed), which both can be very valuable for small, endemic, yet endangered species. We believe that implementation of values of effective population size should be included into the IUCN criterion C, in order to avoid questionable extrapolations from effective to census population sizes [23]. That does not mean complete withdrawal of the census population size values, since they can be very useful when they are obtained either through demographic investigations or information on *N*_*e*_ */N* ratio is available for taxonomic group in question. However, since the mentioned ratio differs greatly among taxa due to differences in demography and life history, effective population size could be used just as effectively, but more correctly for extinction risk assessments using criterion C. For example, if *N*_*e*_ of *C*. *herzegoviniensis* would be extrapolated into *N* using 0.1 value, number of mature individuals would fit it into VU category. However, if we use maximal default value of 0.2 [23], the same effective population size would fall out of the endangerment criteria. The possibility of mistake is even greater if *N*_*e*_/*N* of spined loaches falls out of the currently proposed values, since estimates are available for only about 100 species [23]. Furthermore, our results speak in favor of inclusion of even more population genetic data in extinction risk assessments–since intrapopulational polymorphism is positively correlated with mean fitness, extinction risk could be estimated based on polymorphism measures also and we find justified to include them into the IUCN Red List categorization system, probably as an additional criterion. The objection that lower genetic diversity does not necessarily equate to elevation risk because it is usually the symptom of endangerment and not its cause [50], [51] does not reduce its possible usefulness in the estimation of the extinction risk for which it is a “symptom”. Furthermore, our example of the Adriatic spined loaches demonstrates that small genetic diversity may indeed be the cause of greater extinction risk. Namely, same anthropologic action will probably have more significant impact on *C*. *jadovaensis* and *C*. *herzegoviniensis* than on *C*. *dalmatina* and *C*. *narentana* precisely because of their smaller intraspecific diversity which, on the other hand, was not induced by demographic collapse, but by evolutionary history. Supplementary population genetic information (in particular information on intraspecific genetic structure and possible migration rates and directions) are also extremely useful for defining effective conservation measures and ensuring best possibility of survival for endangered species using financial resources in the most reasonable manner.
